# Curcumin attenuates liver injury by modulating the AGE–RAGE axis and metabolic homeostasis in high-fat diet/streptozotocin-induced type 2 diabetic mice

**DOI:** 10.3389/fnut.2025.1710380

**Published:** 2025-11-07

**Authors:** Mengyao Li, Chunmei Zhang, Junyu Ma, Bangzhao Zeng, Xuexun Li, Xin Zhao, Xiaoyan Bi, Rong Li, Qin Gao, Yang Jiang, Fuli Ya

**Affiliations:** 1 Department of Nutrition, School of Public Health, Dali University, Dali, Yunnan Province, China; 2 Department of Laboratory Teaching Center, School of Public Health, Dali University, Dali, Yunnan Province, China; 3 School of Public Health, Jining Medical University, Jining, Shandong Province, China

**Keywords:** *Curcuma longa*, curcumin, diabetic liver injury, type 2 diabetes mellitus, AGE–RAGE signaling, metabolomics

## Abstract

**Background:**

Diabetic liver injury is a serious complication of type 2 diabetes mellitus (T2DM). Curcumin (CUR), a natural polyphenol derived from *Curcuma longa*, exhibits diverse biological activities. This study investigated the hepatoprotective effect of CUR against liver injury in a high-fat diet/streptozotocin (HFD/STZ)-induced T2DM mouse model and elucidated the underlying mechanisms.

**Methods:**

We integrated network pharmacology to identify common targets between CUR and T2DM, followed by molecular docking to evaluate binding affinities to key targets. *In vivo*, T2DM mice induced by HFD/STZ were administered dietary CUR (800 mg/kg diet) for 4 weeks. Hepatic oxidative stress, inflammatory markers, key signaling pathways, and metabolomic profiles were analysed.

**Results:**

Network pharmacology revealed 256 overlapping targets between CUR and T2DM. Protein–protein interaction (PPI) analysis identified AKT1, TNF, TP53, IL-6, and EGFR as central hub targets. KEGG pathway enrichment suggested the involvement of the advanced glycation end products (AGE)–RAGE signaling pathway in the protective effects of CUR. Molecular docking demonstrated strong binding affinities of CUR with RAGE, AKT1, and TP53. *In vivo*, CUR supplementation significantly improved hyperglycemia and reduced hepatic oxidative stress and inflammation in T2DM mice. CUR inhibited the AGE–RAGE pathway and modulated downstream PI3K/Akt and NF-κB signaling. UPLC–MS/MS-based metabolomics analysis indicated that CUR altered metabolic pathways related to galactose, glycine/serine/threonine, propanoate, and nicotinate/nicotinamide.

**Conclusion:**

CUR protects against diabetic liver injury by inhibiting AGE–RAGE-induced inflammation and metabolic dysregulation. The protective mechanism involves modulation of the AGE–RAGE axis and restoration of metabolic homeostasis.

## 1 Introduction

Type 2 diabetes mellitus (T2DM) has emerged as a critical global public health challenge, with its prevalence escalating rapidly in both developed and developing nations. According to the Lancet Public Health series, the global diabetic population reached 529 million in 2021 and is projected to surge to 1.31 billion by 2050, with China accounting for over 118 million cases (22% of the global burden) (1). Notably, China’s epidemiological profile exhibits unique characteristics, including a narrowing urban–rural prevalence gap, earlier disease onset (increasingly diagnosed before age 40), and a high undiagnosed rate (56% in rural areas) (2). Uncontrolled T2DM imposes a multidimensional burden on human health. It significantly elevates cardiovascular mortality, with diabetic patients facing a 2.5-fold higher risk of myocardial infarction and stroke due to endothelial damage and hypercoagulability (3). Moreover, other complications such as diabetic retinopathy and nephropathy attributed to T2DM are pervasive and significantly increase the burden of non-communicable diseases (NCDs) (4).

Non-alcoholic fatty liver disease (NAFLD) has become an epidemic, much like other NCDs, such as obesity, diabetes, and cardiovascular diseases (5). T2DM is an important risk factor for NAFLD (6). The pathogenesis of T2DM leading to NAFLD is a complex interplay of metabolic dysregulation, insulin resistance (IR), and chronic inflammation. Central to this relationship is IR, which serves as a shared pathophysiological mechanism (7). In T2DM, IR disrupts glucose homeostasis and promotes lipolysis in adipose tissue, releasing excessive free fatty acids (FFAs) into the circulation. These FFAs are transported to the liver, where they overwhelm mitochondrial *β*-oxidation capacity, leading to hepatic triglyceride accumulation and subsequent steatosis (8). Moreover, chronic IR in T2DM triggers adipose tissue inflammation, releasing pro-inflammatory cytokines (e.g., TNF-*α*, IL-6) that impair hepatic insulin signaling and recruit circulating monocytes to the liver (9). Within hepatocytes, lipid overload induces mitochondrial dysfunction and endoplasmic reticulum stress, generating reactive oxygen species (ROS) that activate NFκB pathway, amplifying secretion of inflammatory cytokine (10, 11). Concurrently, hyperglycemia-driven AGEs engage RAGE on hepatocytes, activating downstream pro-inflammatory pathways such as NFκB, exacerbating hepatic inflammation and oxidative stress (12–14). These intertwined mechanisms establish a self-sustaining inflammatory microenvironment that accelerates hepatocyte apoptosis, macrophage infiltration, and progressive NAFLD transition to steatohepatitis (NASH) (6, 7). Thus, pharmacological intervention interfering either directly or indirectly with the AGEs/RAGE axis can prove to be a valid therapeutic approach to prevent the progression of diabetic complications (15).

The primary strategy for halting NAFLD progression to non-alcoholic steatohepatitis (NASH) and hepatocellular carcinoma (HCC) centers on controlling underlying risk factors including diabetes, hyperlipidemia, obesity, and associated comorbidities (16, 17). Dietary intervention represents a cornerstone approach for preventing and attenuating T2DM progression and concomitant NAFLD (18, 19). Curcumin (CUR), a bioactive polyphenol primarily extracted from the dietary spice *Curcuma longa* (turmeric) (20), has demonstrated protective potential for metabolic disorders. CUR exhibits diverse pharmacological activities, including antioxidant, anti-inflammatory, and anticancer effects (21, 22). Our prior studies established that dietary CUR supplementation significantly attenuated allergic asthma in murine models (23). We also showed that treatment of CUR greatly attenuated murine thrombus growth in a FeCl_3_-induced mesenteric arteriole thrombosis in C57BL/6J mice (24). Moreover, CUR plays important protective roles in NAFLD (25, 26). Nevertheless, the mechanistic basis for the efficacy of CUR against diabetic liver injury remains incompletely characterized. This study therefore integrates network pharmacology, molecular docking, experimental validation, and untargeted metabolomics to investigate the hepatoprotective effects of CUR and elucidate the underlying mechanisms in T2DM mice.

## 2 Materials and methods

### 2.1 Network pharmacology analysis

#### 2.1.1 Curcumin target prediction

The chemical structure, Chemical Abstracts Service registry number, and Simplified Molecular Input Line Entry System notation of CUR were retrieved from PubChem.^1^ Putative targets were identified using SwissTargetPrediction,^2^ TargetNet,^3^ SuperPred,^4^ and Search Tool for Interactions of Chemicals (see footnote 4).

#### 2.1.2 Acquisition of T2DM-associated targets

T2DM-related targets were curated from Online Mendelian Inheritance in Man,^6^ Therapeutic Target Database,^7^ GeneCards,^8^ PharmGKB,^9^ and MalaCards^10^ databases.

#### 2.1.3 Protein–protein interaction (PPI) network construction

Overlapping targets between CUR and T2DM were determined using a Venn diagram tool.^11^ These targets were subsequently imported into Search Tool for the Retrieval of Interacting Genes/Proteins (*Homo sapiens*; medium confidence score > 0.400) to construct a PPI network, which was analysed with Cytoscape.^12^

#### 2.1.4 Identification of core targets

The PPI network was visualized in Cytoscape 3.9.1. Six topological parameters (Degree Centrality, Betweenness Centrality, Closeness Centrality, Eigenvector Centrality, Network Centrality, and Local Average Connectivity) were computed using the Network Analyzer and CytoNCA plugins. Core therapeutic targets were identified through two-tiered median-based filtering of these metrics. CytoHubba was then employed to rank the top 30 core genes based on Maximal Clique Centrality, Maximum Neighborhood Component, and high-confidence interactions. Key targets were derived from the intersection of these results.

#### 2.1.5 Functional enrichment analysis

Overlapping targets were submitted to the DAVID database for GO and KEGG pathway enrichment (*p* < 0.01). The top 10 enriched terms in biological processes (BP), cellular components (CC), molecular functions (MF), and signaling pathways were visualized using ClueGo in Cytoscape 3.9.1 through bar charts and bubble plots.

#### 2.1.6 Compound-target-pathway (C-T-P) network construction

A tripartite network integrating CUR, predicted targets, and enriched pathways was constructed in Cytoscape 3.9.1. Nodes represent molecular entities while edges denote biological associations, enabling systematic prediction of CUR’s therapeutic mechanisms in T2DM.

#### 2.1.7 Molecular docking analysis

Molecular docking was performed between CUR and the top 5 PPI-ranked core targets, along with RAGE. The 3D structure of CUR was obtained from PubChem, while the structures of the target proteins were retrieved from the RCSB PDB.^13^ Both ligand and protein structures were preprocessed using Open Babel.^14^ The interactions between the ligands and receptors, as well as the binding affinities (−CDOCKER_ENERGY/−CDOCKER_INTERACTION_ENERGY, where higher values indicate stronger binding), were calculated using the precise docking program CDOCKER in Discovery Studio 2017 R2 Client. The resulting docking poses were visualized using PyMOL.

### 2.2 Chemicals and reagents

CUR (purity ≥ 98%; CAT#C805205) was purchased from Macklin (Shanghai, China). Antibodies against PI3K (CAT#AF6241), phospho-PI3K (Tyr^607^) (CAT#AF3241), Akt (CAT#AF6261), phospho-Akt (Ser^473^) (CAT#AF0016), phospho-NFκB p65 (Ser^536^) (CAT#AF2006), TNF-*α* (CAT#AF7014), and IL-1*β* (CAT#AF5103) were purchased from Affinity Biosciences (OH, USA). Antibodies against NFκB p65 (CAT#BS1257) and β-actin (CAT#AP0060) were purchased from Bioworld Technology (MN, USA). Antibody against IL-6 (CAT#GB11117) and secondary antibodies (goat anti-rabbit) (CAT#GB23303) were purchased from Servicebio (Wuhan, China). Streptozotocin (STZ, purity ≥ 85%; CAT#S8050) was purchased from Solarbio (Beijing, China).

### 2.3 Animals and diets

**Figure 1:**
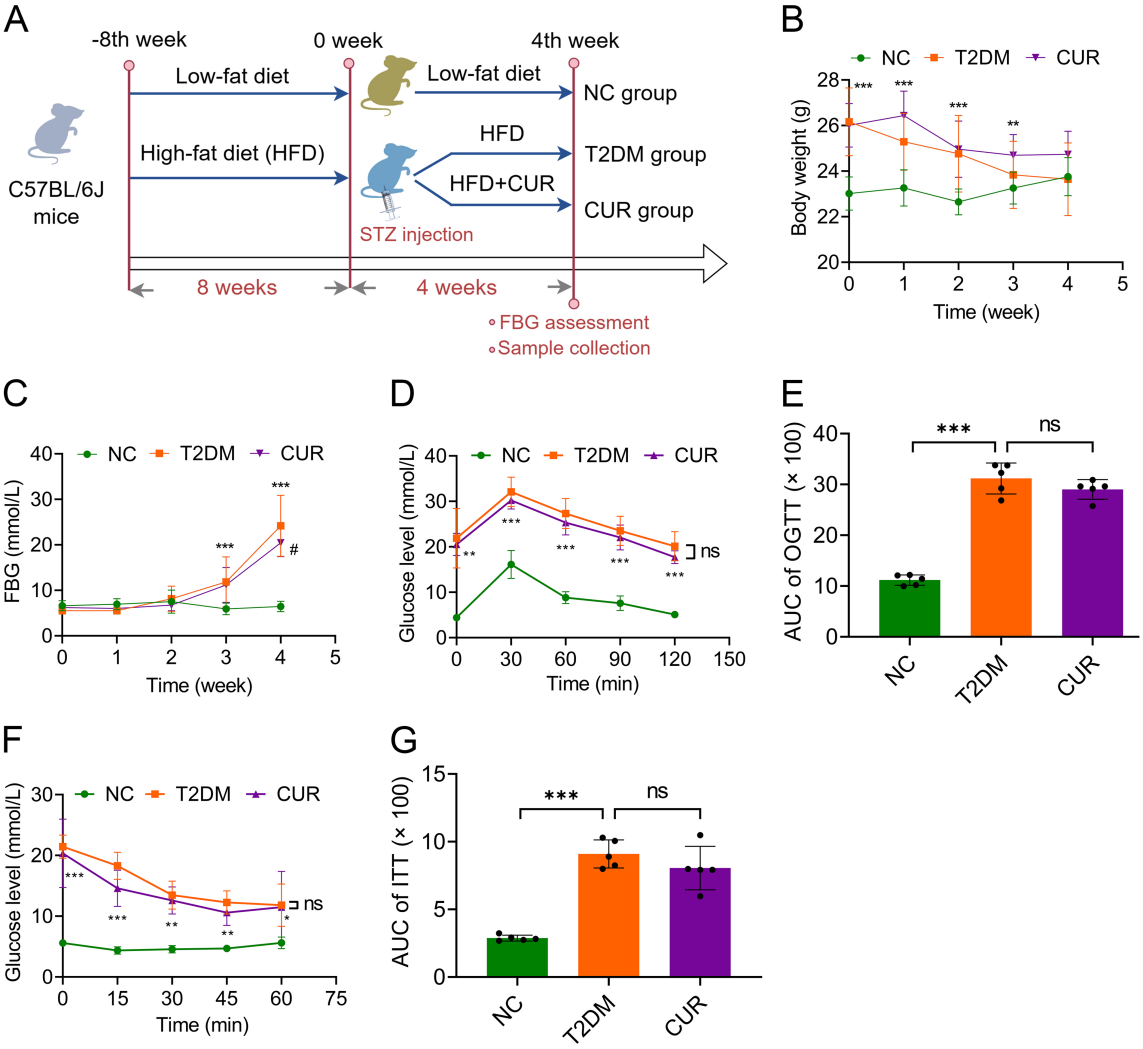
CUR supplementation improves FBG in T2DM mice. **(A)** Flowchart of the *in vivo* experiments was shown. **(B)** The body weight of mice was monitored in the NC, T2DM, and CUR groups (*n* = 10). **(C)** The weekly FBG levels were shown (*n* = 10). **(D,E)** The blood glucose levels were measured in the OGTT assays **(D)** and the quantitative trapezoidal AUC analysis of OGTT was presented (*n* = 5) **(E)**. **(F,G)** The blood glucose levels were measured in the ITT assays **(F)** and the quantitative trapezoidal AUC analysis of ITT was presented (*n* = 5) **(G)**. **p < 0.01 and ***p < 0.001: NC group *vs.* T2DM group; ^#^*p* < 0.05: T2DM *vs.* CUR group; ns, not significant difference.

A total of 30 male C57BL/6J mice (aged 7 weeks) were obtained from Henan Skobes Biotechnology Co., Ltd. (Zhengzhou, Henan, China). The animals were raised in the specific-pathogen-free animal lab, and housed under standard environmental conditions with a ambient temperature maintained at 22 ± 2 °C and photoperiod synchronization maintained under a controlled 12-h light/dark cycle (lights on at 7:00 a.m.). Following a 7-day acclimation period with standard low-fat diet (LFD), the animals were randomly divided into two groups and fed either the LFD (NC group, *n* = 10; containing 10% fat) or a high-fat diet (HFD group, *n* = 20; containing 45% fat) for 8 weeks. Following a 12-h fasting period (water ad libitum), the animals in HFD group were further randomly divided into T2DM induction group (*n* = 10) and CUR intervention group (CUR group, *n* = 10). These two groups received weekly intraperitoneal injections of streptozotocin (STZ, 80 mg/kg body weight) dissolved in 0.1 mol/L citrate buffer (pH 4.5) for four consecutive weeks, while the NC group was administered equivalent volumes of sterile citrate buffer. Throughout the 4-week induction period, dietary regimens were maintained as follows: NC group received the LFD, while T2DM induction group and CUR group were provided with the HFD and the HFD supplemented with CUR (800 mg/kg diet) through precision feed-mixing technology, respectively. Animal feeds were purchased from Medicience Ltd. (Jiangsu, China) and the feed composition was shown in Supplementary Table S1. The weight, food intake and fasting blood glucose (FBG) levels were monitored weekly. The FBG levels of all mice in T2DM induction group were ≥11.1 mmol/L (200 mg/dL); this indicated that the T2DM model has been successfully established and set as T2DM group. The schematic diagram for the modeling process is shown in Figure 1A. All experiments were approved by the Dali University Animal Care and Use Committee of Dali University (permit number: SYXK [Dian] 2018-0002).

### 2.4 Oral glucose tolerance test (OGTT) and insulin tolerance tests (ITT)

Randomly selected mice were fasted for 16 h with free access to water prior to the OGTT experiment. Body weight was recorded, followed by oral administration of 20% (w/v) filter-sterilized glucose solution using autoclaved stainless steel feeding needles at a dose of 2 g/kg body weight. Mice were restrained using specialized rodent holders, and the tail tip was disinfected with 75% ethanol. After puncturing the tail vein with a sterile lancet, the first blood droplet was wiped away using sterile gauze. Subsequent blood samples were collected for baseline glucose measurement using glucose test strips and a calibrated glucometer (Sinocare, China). Blood glucose levels were monitored at 0 (baseline glucose levels), 30, 60, 90, and 120-min post-administration intervals. The glucose excursion curve was plotted against time, and the area under the curve (AUC) was calculated using the trapezoidal method. The ITT was performed as previously described. Briefly, after measuring the baseline glucose levels, the mice were fasted for a period of 8 h and then injected intraperitonealy with insulin (0.75 U/kg body weight). Glucose levels in tail blood were determined at 15, 30, 45 and 60 min after the insulin injection and AUC was then calculated.

### 2.5 Blood and liver tissue collection

The study was conducted in accordance with the ARRIVE guidelines and all methods were performed following the American Veterinary Medical Association (AVMA) Guidelines for the Euthanasia of Animals (2020). The principles established in the institutional guidelines were faithfully followed during the duration of this study. Mice were first deeply anesthetized with isoflurane, followed by an intraperitoneal injection of a lethal overdose of pentobarbital sodium (150 mg/kg body weight) to ensure death. Upon confirming the loss of pedal and other reflexes, blood was collected via cardiac puncture until death was confirmed. No signs of peritonitis or significant abdominal distress were observed at the time of injection or during the subsequent dissection.

Fasting blood glucose (FBG) in the blood samples collected via tail vein puncture was immediately measured using a calibrated glucometer. Then, blood was collected following cardiac puncture and drawn into tubes containing 1/10 volume of 3.8% acid citrate dextrose (38 mM citric acid, 75 mM trisodium citrate, 100 mM dextrose). Next, the anticoagulated blood was centrifugated at 300×*g* for 2 min at 37 °C to obtain platelet-rich plasma (PRP). PRP was further centrifugated at 500×*g* for 3 min and the supernatant was then transferred into a new tube and stored at −80 °C until further analysis. After collecting blood samples, the vasculature was flushed with saline through the left ventricle to flush the blood out of the mice after the thoracic cavity was opened. Then, liver tissue was collected and weighted.

### 2.6 Hematoxylin–eosin (HE) staining

HE staining was performed as previously describe (27–29). Briefly, freshly harvested liver was fixed in 4% paraformaldehyde (PFA, pH 7.2) and embedded in paraffin. Liver sections (5 μm in thickness) were stained with HE for morphological evaluation. Sections were digitalized using a Leica microscope (Leica Microsystems, Heidelberg, Germany).

### 2.7 Measurement of plasma aspartate aminotransferase (AST) and alanine aminotransferase (ALT)

The levels of ALT (CAT#AKAM006M) and AST (CAT#AKAM019M), two basic liver function biomarkers, were determined at the plasma using commercially available assay kits according to the manufacturer’s instructions (Beijing Boxbio Science & Technology Co., Ltd., Beijing, China). Briefly, 5 μL of sample supernatant was sequentially added to each well, followed by the working reagent. After a two-step incubation process, 250 μL of chromogenic reagent was dispensed into each well to initiate color development. The plate was gently mixed and incubated at room temperature for 10 min, after which the optical density (OD) of each well was measured at 505 nm using a microplate reader.

### 2.8 Measurement of total antioxidant capacity (T-AOC), superoxide dismutase (SOD) activity, and malonaldehyde (MDA)

Levels of T-AOC (CAT#AKAO012M), SOD activity (CAT#AKAO001M) and MDA (CAT#AKFA013M) in the liver tissue were measured using a T-AOC Assay Kit, SOD Activity Assay Kit, and MDA Content Assay Kit (Beijing Boxbio Science & Technology Co., Ltd., Beijing, China), respectively. Briefly, the homogenization of liver tissue (100 mg) was performed in an ice bath for 15 min and centrifugated at 10,000×*g* for 10 min at 4 °C to remove insoluble material. The supernatant was then transferred into a fresh tube, incubated on ice, and the protein concentrations were determined. Finally, T-AOC, SOD activity and MDA were determined according to the manufacturer’s instruction. The absorbance was read in a microplate reader at awavelength of 593 nm for T-AOC, 560 nm for SOD activity, 450, 532 and 600 nm for MDA. T-AOC, SOD activity and MDA levels were calculated and expressed in μmol/mg protein, units/mg protein and nmol/mg protein, respectively.

### 2.9 Quantitative reverse transcriptase real-time polymerase chain reaction (qRT-PCR)

Total RNA was extracted from the liver tissues using Animal tissue total RNA extraction kit (Servicebio, Wuhan, China; CAT#G3640) according to the manufacturer’s instructions. After RNA isolation, 1 μg of total RNA was reverse transcribed to cDNA using an SweScript All-in-One RT SuperMix for qPCR (Servicebio, Wuhan, China; CAT#G3337). Primer sequences for all genes (IL-1*β*, IL-6, TNF-*α*, and RAGE) were listed in Supplementary Table S2. The housekeeping gene β-actin was used as an internal control for quantification. Quantitative real-time PCR was performed using the SuperReal PreMix Plus (TianGen Biotech Co., Ltd., Beijing, China; CAT#FP205-02) on the QuantStudio 3 Real-Time PCR System (Thermo Fisher Scientific, MA, USA). Relative mRNA expression of target genes was then calculated using the 2-ΔΔCt method.

### 2.10 Measurement of AGEs levels in the plasma and liver tissues

The AGEs levels in the plasma and liver tissues were determined using a mouse-specific ELISA kit (CUSABIO Biotech, Wuhan, China; CAT#CSB-E09414m) according to the manufacturer’s protocol. The liver tissues were lysed using a tissue homogenizer (Servicebio, Wuhan, China), followed by centrifugation at 5,000×g for 5 min at 4 °C. The supernatant was transferred into a fresh tube and the protein concentrations were determined using a BCA Protein Assay Kit (Beyotime Biotechnology, Shanghai, China; CAT#P0011). The AGEs levels in the supernatant were determined according to the manufacturer’s instruction. The absorbance was read in a microplate reader at awavelength of 450 nm. The AGEs levels were calculated and expressed in μg/mL for the plasma and μg/mg protein for the liver tissues, respectively.

### 2.11 Western blotting analysis

The liver tissues were homogenized in ice-cold RIPA buffer (25 mM Tris–HCl pH 7.6, 150 mM NaCl, 1% Triton X-100) supplemented with protease/phosphatase inhibitor cocktail (Beyotime Biotechnology Corporation, Shanghai, China) using a tissue homogenizer at 4 °C. Lysates were clarified by sequential centrifugation at 3,000×*g* for 10 min to remove cellular debris, followed by ultracentrifugation at 12,000×*g* for 15 min at 4 °C to obtain cytoplasmic fractions. Protein quantification was performed via bicinchoninic acid assay (Beyotime Biotechnology Corporation, Shanghai, China) with bovine serum albumin calibration standards. Aliquots containing 30 μg denatured protein (95 °C for 5 min in Laemmli buffer) were resolved through 10% Tris-glycine SDS-PAGE and electrotransferred onto 0.45 μm PVDF membranes (Millipore; MA, USA) by electroblotting. Western blotting was performed using our previously described methods (24, 30–32). Briefly, following blocking with 5% BSA for 1.5 h, the PVDF membrane was incubated overnight at 4 °C with primary antibodies against the following targets (dilutions in parentheses): PI3K (1:2,000), phospho-PI3K (Tyr^607^) (1:1,000), Akt (1:2,000), phospho-Akt (Ser^473^) (1:1,000), phospho-NFκB p65 (Ser^536^) (1:1,000), NFκB p65 (1:1,000), TNF-*α* (1:500), IL-1*β* (1:1,000), IL-6 (1:1,000), and β-actin (1:20,000) at 4 °C overnight and then washed 3 times in TBST. Subsequently, the membrane was washed three times with TBST and then incubated for 1.5 h with HRP-conjugated goat anti-rabbit IgG (1:20,000) secondary antibodies. After three additional TBST washes, protein bands were visualized using an ECL Western blotting detection system. Band densitometry was performed using ImageJ software (version 1.37v; NIH, USA). Relative protein expression was normalized to β-actin and expressed as fold-change versus NC groups.

### 2.12 Statistical analysis

Data were collected from at least three independent mice and represented as means with standard deviation (mean ± SD). One-way analysis of variance (ANOVA) followed by Tukey’s multiple comparisons test was conducted to determine the statistical significance using GraphPad Prism software (version 9.4.1; GraphPad Inc., San Diego, CA, USA). Differences between groups were considered statistically significant at *p <* 0.05.

### 2.13 Untargeted metabolomics analysis by UPLC–MS/MS

#### 2.13.1 Sample processing procedure

Liver tissue specimens (50.0 ± 0.5 mg wet weight) were cryogenically homogenized in 500 μL ice-cold methanol:water (80:20, v/v) containing 1.0 mm zirconium oxide beads using a high-throughput homogenizer at 6,800 rpm. The homogenates underwent protein precipitation at −20 °C for 30 min followed by sequential centrifugation: primary clarification at 20,000×*g* for 10 min at 4 °C, then secondary centrifugation of the supernatant under identical conditions to remove residual particulates. The final supernatant was filtered through 0.22 μm PTFE membrane (Millipore; MA, USA) and aliquoted into pre-chambered LC-MS vials. For system suitability evaluation, quality control (QC) samples were generated by pooling equal volumes (10 μL) from all experimental specimens followed by 10-min vortex mixing and additional centrifugation at 15,000×*g* for 5 min. All processed samples were maintained at −80 °C in nitrogen-filled cryovials until UPLC-HRMS analysis (within 24 h).

#### 2.13.2 UPLC–MS/MS conditions

For chromatography, An ACQUITY UPLC HSS T3 column (100 mm × 2.1 mm, 1.8 μm, Waters) was used for separation. The mobile phase consists of phase A (5 mmol/L ammonium acetate + 5 mmol/L acetic acid + water) and phase B (acetonitrile). Gradient elution conditions were set as follows: 0–0.8 min, 2% B; 0.8–2.8 min, 2%–70% B; 2.8–5.6 min, 70%–90% B; 5.6–6.4 min, 90%–100% B; 6.4–8.0 min, 100% B; 8.0–8.1 min, 100%–2% B; 8.1–10.0 min, 2% B. The flow rate is 0.35 mL/min. The injection volume for each sample was 4 μL. The column oven was maintained at 40 °C.

For mass spectrometry, a high-resolution tandem mass spectrometer Triple TOF 6600 (AB SCIEX) was used to detect metabolites eluted form the column. Each sample was operated in both positive and negative electrospray ionization (ESI) mode. ESI temperature is 500 °C. The voltage is +5,000 volts in ESI^+^ mode and −4,500 volts in ESI^−^ mode. The curtain gas pressure of the ion source is 30 psi, Gas 1 (Auxiliary gas) and Gas 2 (Sheath gas) pressures are both set to 60 psi. The mass spectrometric data were obtained with full scan and information dependent acquisition (IDA) modes. In one acquisition cycle, the full scan acquisition range is 60–1,200 Da, and the full scan acquisition time is 150 ms. Then, the top 12 signal ions with a signal accumulation intensity of more than 100 were selected from the full scan for IDA scanning, the IDA acquisition range is 25–1,200 Da, and the acquisition time is 30 ms. Dynamic exclusion is set to 4 s.

## 3 Results

### 3.1 CUR supplementation improves FBG in T2DM mice

Given that diabetic liver injury is a frequent complication of T2DM, we examined the protective role of CUR supplementation against liver injury and its underlying mechanisms in T2DM mice (Figure 1A). As shown in Figure 1B, mice fed an HFD exhibited significantly higher body weight than the NC group by the end of the 8th week. However, STZ administration resulted in a significant decrease in body weight. Although CUR supplementation did not favorably restore body weight to normal levels, a trend towards improvement was observed (Figure 1B). Moreover, after 4 weeks of HFD combined with STZ administration, FBG levels in the T2DM model group (24.19 ± 6.69 mmol/L) were significantly higher than those in the control group (6.44 ± 1.15 mmol/L) (Figure 1C). Following 4 weeks of CUR supplementation, FBG levels were both substantially prevented from rising further and partially reversed compared to the HFD/STZ-induced increase (Figure 1C).

We further employed OGTT to assess insulin resistance. The T2DM group exhibited marked hyperglycemic excursions, peaking at 30 min post-challenge with attenuated clearance kinetics (Figure 1D). However, CUR supplementation failed to significantly attenuate acute hyperglycemia or accelerate monophasic glucose decay (Figure 1D). Quantitative trapezoidal area under the curve (AUC) analysis confirmed no significant reduction in glycemic exposure with CUR intervention versus the T2DM group (Figure 1D) Similar results were observed in the ITT, an assay assessing systemic insulin responsiveness (Figures 1F,G).

### 3.2 CUR ameliorates liver injury in T2DM mice

**Figure 2:**
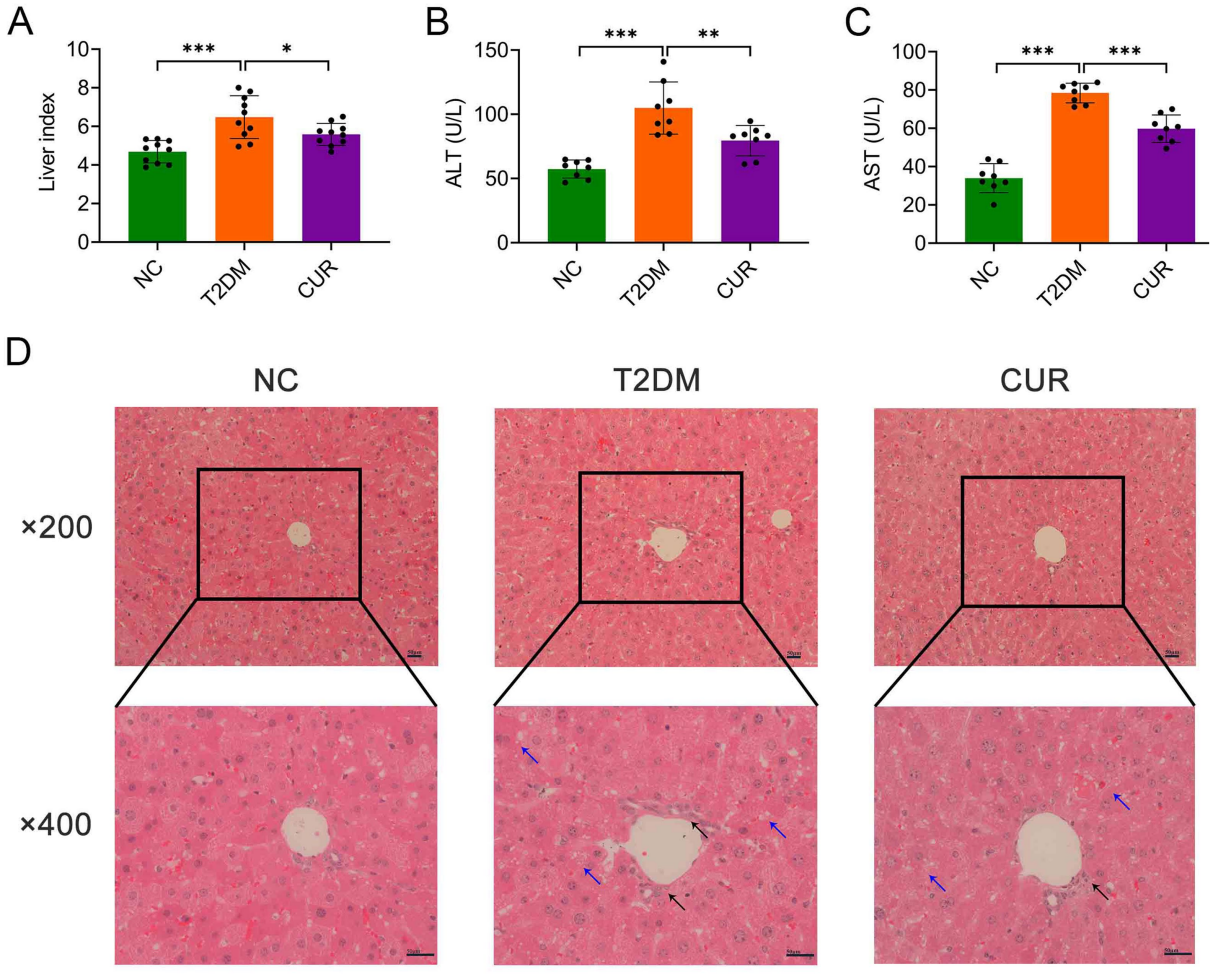
CUR ameliorates liver injury in T2DM mice. **(A)** Liver index was calculated as liver weight/body weight (*n* = 10). **(B,C)** Plasma levels of ALT **(B)** and AST **(C)** were measured (*n* = 8). *p < 0.05, **p < 0.01 and ***p < 0.001. **(D)** Representative images from HE staining of the liver were presented (*n* = 3). Overview (upper panel, ×200 magnification) and detailed views (lower panel, ×400 magnification) were shown. Black arrows denoted inflammatory cell infiltrates, while blue arrows indicated hepatocellular steatosis characterized by cytoplasmic microvesicular vacuolization. Scale bars: 50 μm.

As shown in Figure 2A, the liver index was significantly elevated in the T2DM group compared to NC controls, while CUR supplementation effectively reduced this parameter. Furthermore, plasma levels of ALT and AST were markedly increased in the T2DM group relative to NC group, which were significantly attenuated by CUR supplementation (Figures 2B,C). Histopathological analysis of HE-stained liver sections revealed normal hepatic architecture in NC controls (Figure 2D). In contrast, T2DM mice exhibited significant pathological alterations including lipid vacuolation, steatosis, and inflammatory cell infiltration. These structural abnormalities were substantially ameliorated by CUR intervention (Figure 2D).

### 3.3 CUR attenuates hepatic oxidative stress and inflammation in T2DM mice

**Figure 3:**
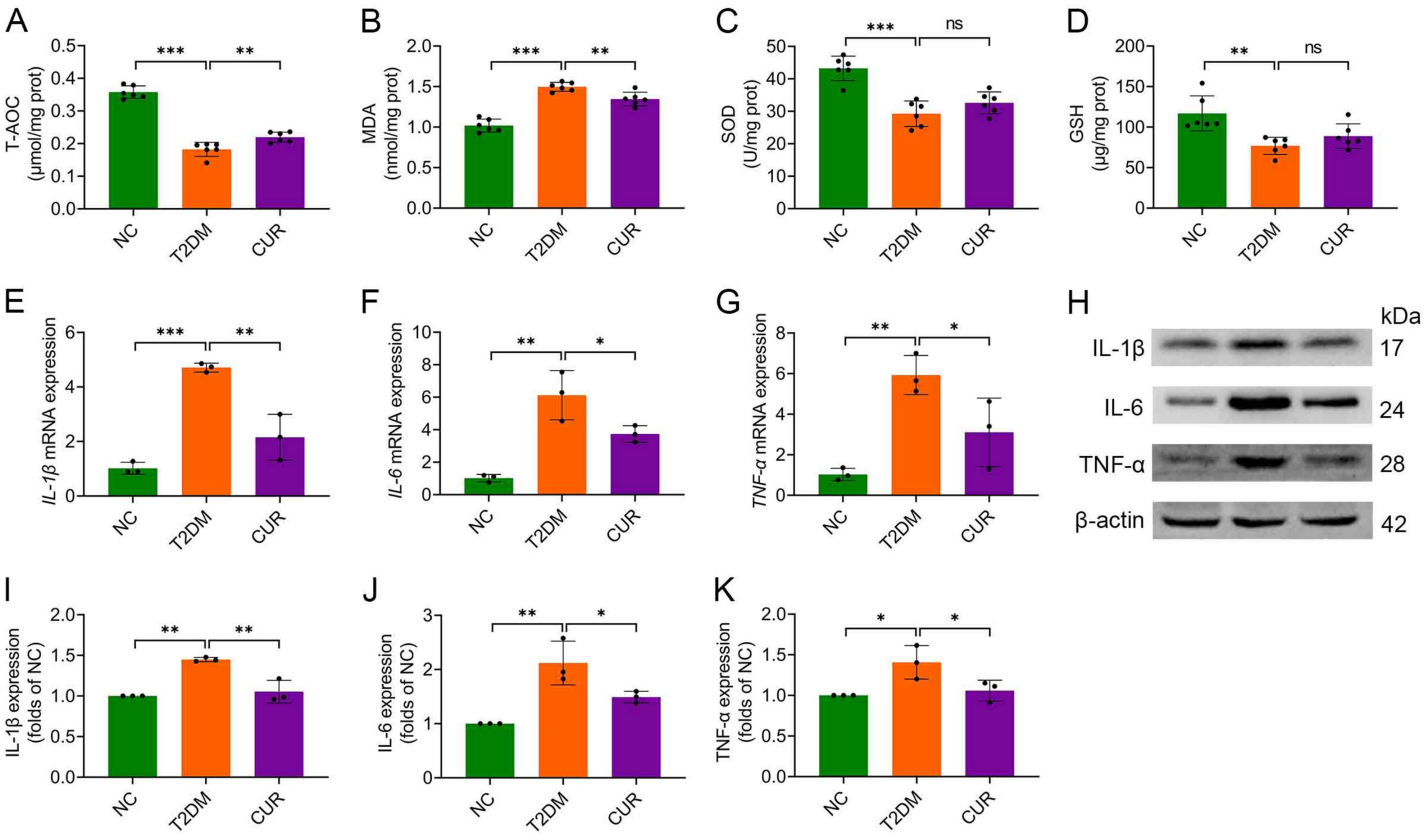
CUR attenuates hepatic oxidative stress and inflammation in T2DM mice. **(A–D)** Hepatic levels of T-AOC **(A)**, MDA **(B)**, SOD activity **(C)**, and GSH **(D)** were determined (*n* = 6). **(E–G)** Hepatic mRNA expression levels of *IL-1β* **(E)**, *IL-6* **(F)**, and *TNF-α* **(G)** were analysed (*n* = 3). **(H)** Representative Western blot bands of hepatic IL-1β, IL-6, and TNF-α proteins. β-actin served as loading control. **(I–K)** Quantitative hepatic protein expression levels of IL-1β **(I)**, IL-6 **(J)**, and TNF-α **(K)** were analysed (*n* = 3). For quantitative analysis **(I–K)**, the band intensity of each target protein was normalized to its corresponding internal control. The normalized values were presented relative to the mean of the NC group, which was set to 1. *p < 0.05, **p < 0.01 and ***p < 0.001; ns, not significant difference.

We next assessed the impact of CUR supplementation on hepatic oxidative stress and inflammation. Compared with NC controls, T2DM mice exhibited significantly reduced hepatic T-AOC, which was partially restored by CUR intervention (Figure 3A). CUR treatment also substantially attenuated T2DM-elevated MDA levels in liver tissue (Figure 3B). Although an upward trend was observed, 4-week CUR supplementation failed to significantly reverse T2DM-induced reductions in SOD and GSH levels (Figures 3C,D). Furthermore, qRT-PCR and immunoblotting analyses demonstrated significant upregulation of pro-inflammatory cytokines (IL-1β, IL-6, TNF-*α*) at both transcriptional (Figures 3E–G) and translational (Figures 3H–K) levels in T2DM livers versus NC controls. CUR supplementation significantly attenuated these inflammatory responses (Figures 3E–K).

### 3.4 Network pharmacology analysis

**Figure 4:**
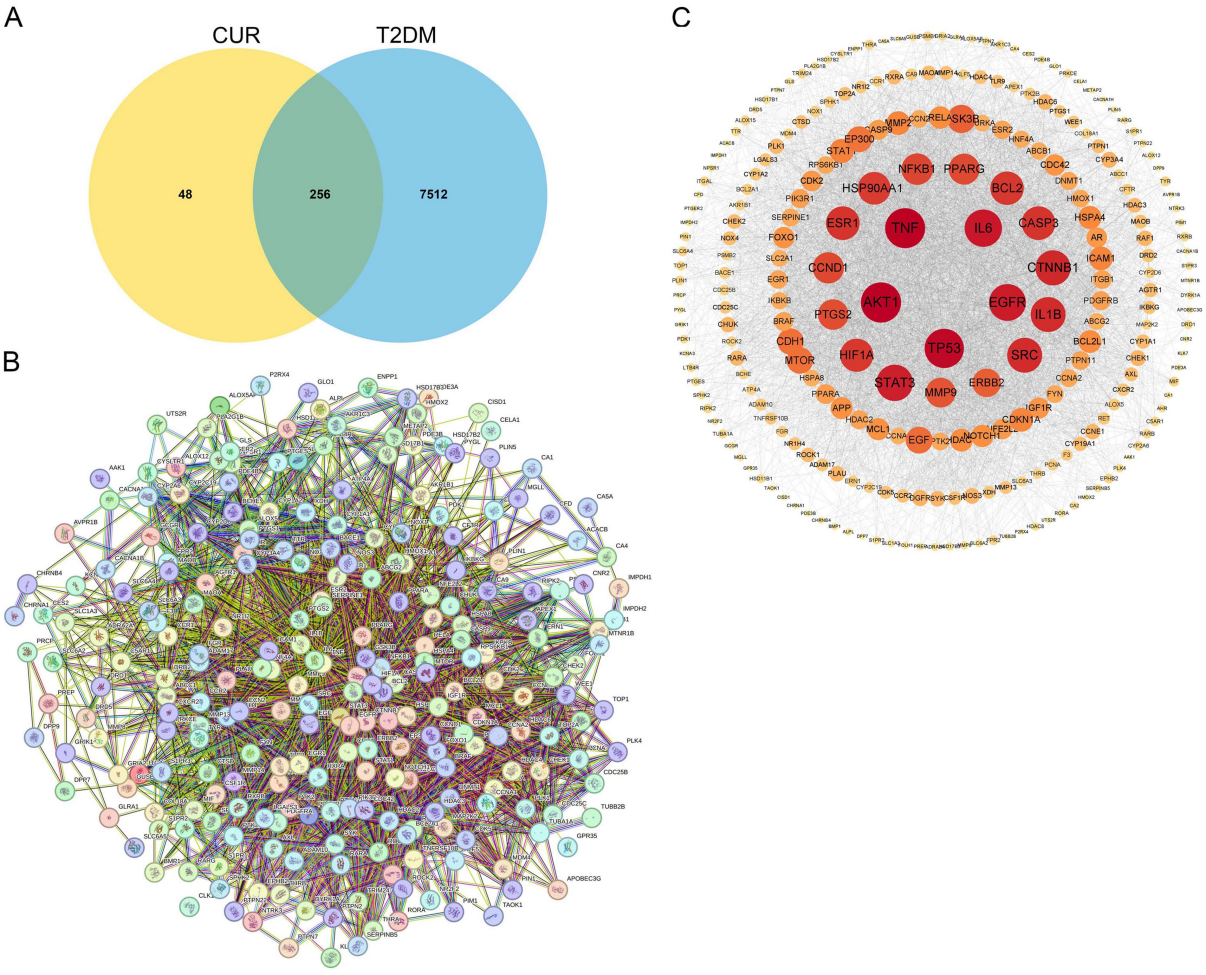
Identification of hub genes through PPI network analysis. **(A)** Venn diagram of the intersection of the CUR and T2DM target genes. **(B)** The PPI network of the 256 common targets was constructed using the STRING database. Each node represents a relevant gene, and the edge thickness indicates the strength of the data support. **(C)** Proteins within the PPI network were ranked by their degree centrality.

Using the SwissTargetPrediction, TargetNet, SuperPred, and STITCH databases, we identified 304 potential CUR targets. Similarly, querying the OMIM, TTD, GeneCards, PharmGKB, and MalaCards databases yielded 7,768 targets associated with T2DM. These 304 CUR targets and 7,768 T2DM targets were then imported into the Venny 2.1.0 online platform for intersection analysis. This analysis revealed 256 overlapping targets, suggesting 256 potential targets through which CUR may treat T2DM (Figure 4A). The PPI network of the 256 common targets was constructed using the STRING database. The final PPI network, constructed after excluding two targets lacking sufficient interaction data in the STRING database, comprised 254 nodes and 4,291 edges, with an average node degree centrality of 33.79 (Figure 4B). In this network representation, nodes represent target proteins and edges represent interactions between them. Proteins within the PPI network were ranked by their degree centrality (Figure 4C). The analysis demonstrated that the therapeutic targets of CUR exhibited characteristics of multiple networks and synergistic interactions. Topological analysis identified protein kinase AKT-1 (AKT1), tumor necrosis factor (TNF), tumor protein P53 (TP53), interleukin 6 (IL-6), and epidermal growth factor receptor (EGFR) as occupying core positions within the PPI network, indicating their role as hub genes in the action of CUR against T2DM (Figure 4C).

**Figure 5:**
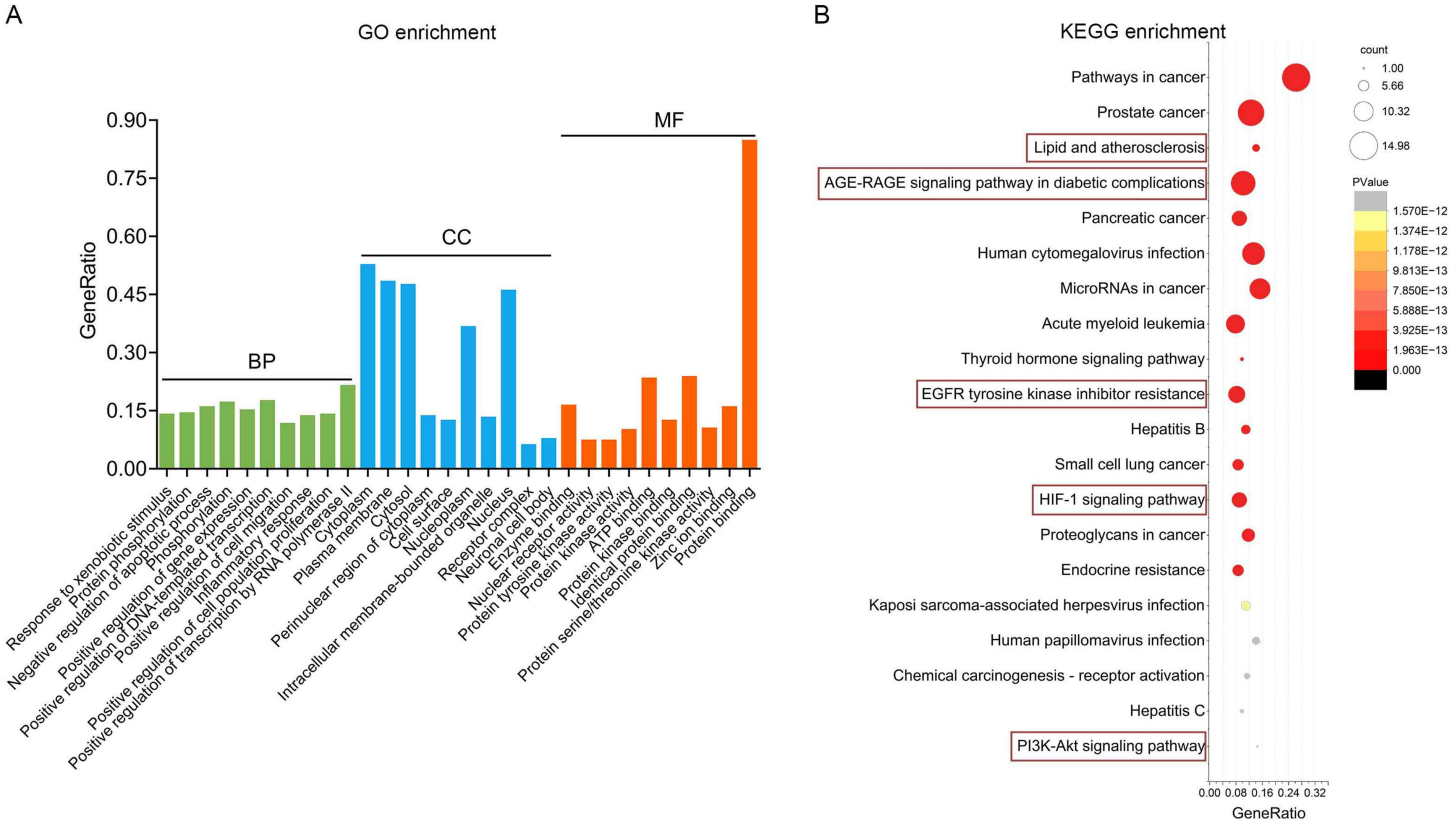
Functional enrichment analysis implicates the AGE–RAGE pathway. **(A)** The top 10 BP, MF and CC in GO enrichment analysis were shown. **(B)** Bubble chart of the top 20 KEGG pathways.

**Figure 6:**
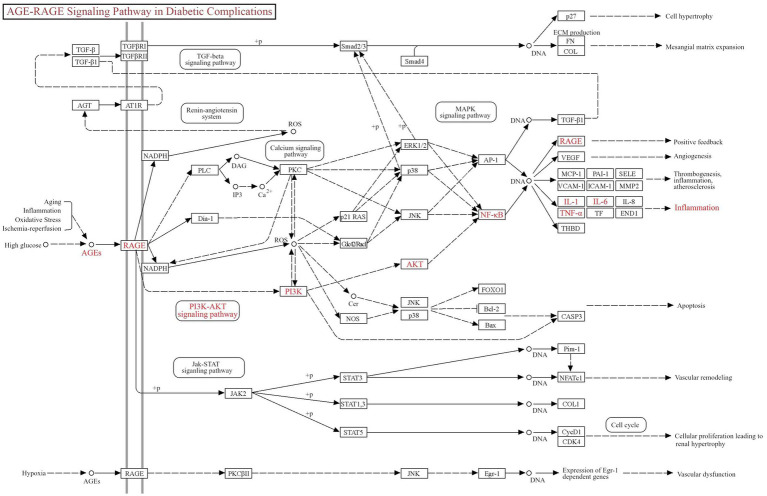
Schematic of the AGE–RAGE signaling pathway in diabetic complications.

GO and KEGG pathway enrichment analyses were performed with a false discovery rate (FDR) threshold of <0.01 for statistical significance. We identified 615 significantly enriched GO terms, comprising 430 BP, 70 CC, and 115 MF. KEGG pathway analysis revealed 168 significantly enriched pathways. The most prominently enriched pathways involved in metabolic disorders, inflammatory damage related functions included AGE–RAGE signaling pathway in diabetic complications, EGFR tyrosine kinase inhibitor resistance, HIF-1 signaling pathway, lipid and atherosclerosis, and PI3K-Akt signaling pathway. Based on FDR and GeneRatio, the top 10 GO entries and top 20 KEGG pathways were shown in Figures 5A,B, respectively. A tripartite network integrating CUR, predicted targets, and enriched pathways was constructed in Cytoscape, as shown in Supplementary Figure S1. KEGG enrichment analysis from our network pharmacology highlighted the AGE–RAGE signaling pathway in diabetic complications as the most relevant. This pathway orchestrates sustained oxidative stress and inflammatory responses through RAGE-mediated PI3K/Akt signaling and NFκB nuclear translocation (Figure 6).

### 3.5 Molecular docking analysis

**Figure 7:**
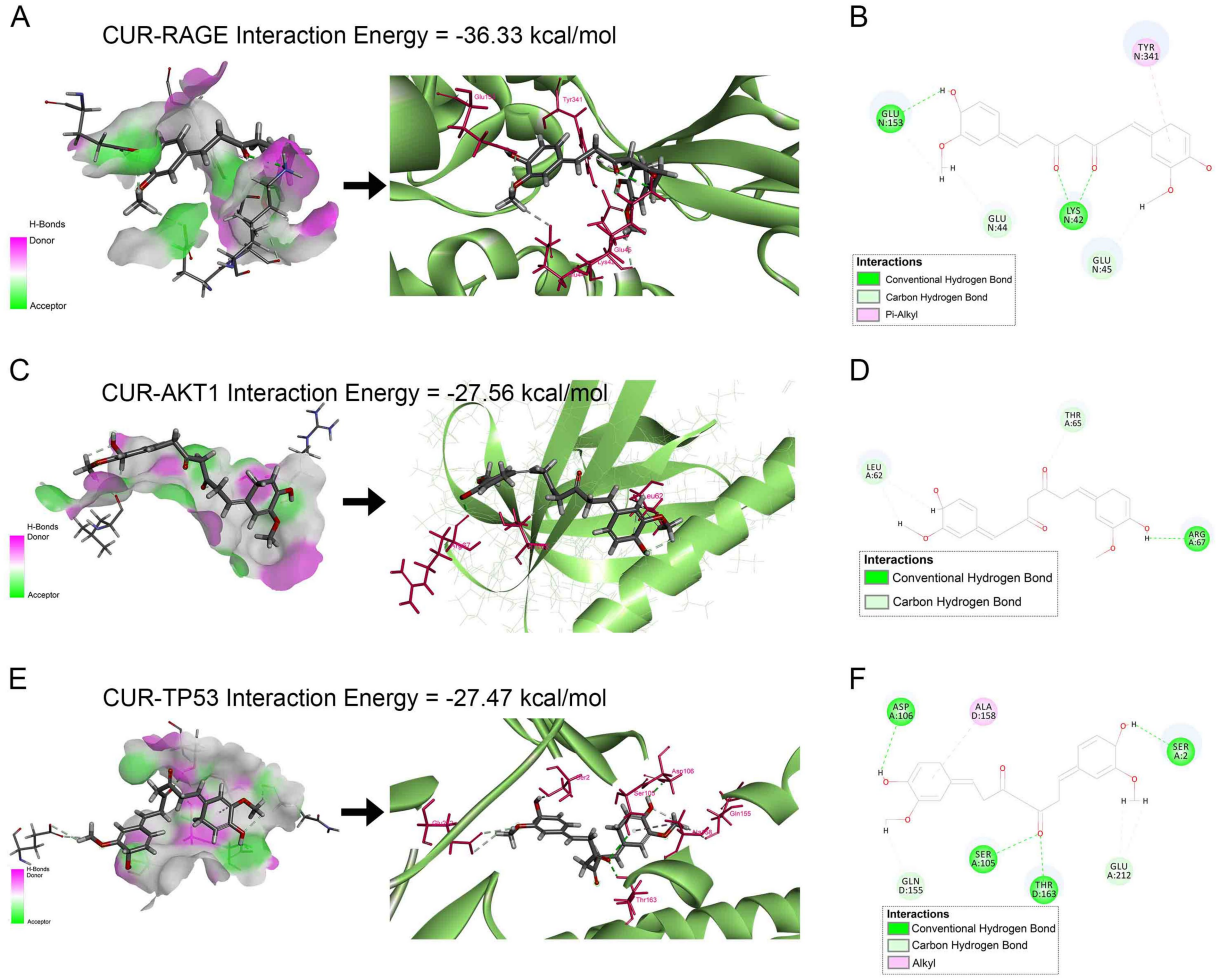
Molecular docking analysis of CUR binding to RAGE, AKT1, and TP53. **(A,C,E)** Left panels: Receptor surface diagrams showing hydrogen bond interactions. Right panels: 3D binding poses of CUR with target proteins. **(B,D,F)** Corresponding 2D ligand interaction diagrams. **(A,B)** CUR-RAGE complex (Binding energy: −36.33 kcal/mol). **(C,D)** CUR-AKT1 complex (Binding energy: −27.56 kcal/mol). **(E,F)** CUR-TP53 complex (Binding energy: −27.47 kcal/mol).

Molecular docking was performed to assess the binding affinity of CUR with the top five PPI-ranked core targets (AKT1, TNF, TP53, IL-6, EGFR) and RAGE. The results demonstrated that CUR exhibited favorable binding affinities towards RAGE, AKT1, and TP53, with docking scores (binding energies) of −36.33, −27.56, and −27.47 kcal/mol, respectively. The corresponding molecular docking poses were presented in Figure 7. However, no significant binding affinity of CUR was observed for TNF, IL-6, or EGFR (data not shown).

### 3.6 CUR modulates AGE/RAGE signaling and its downstream target PI3K/Akt and NFκB pathways

**Figure 8:**
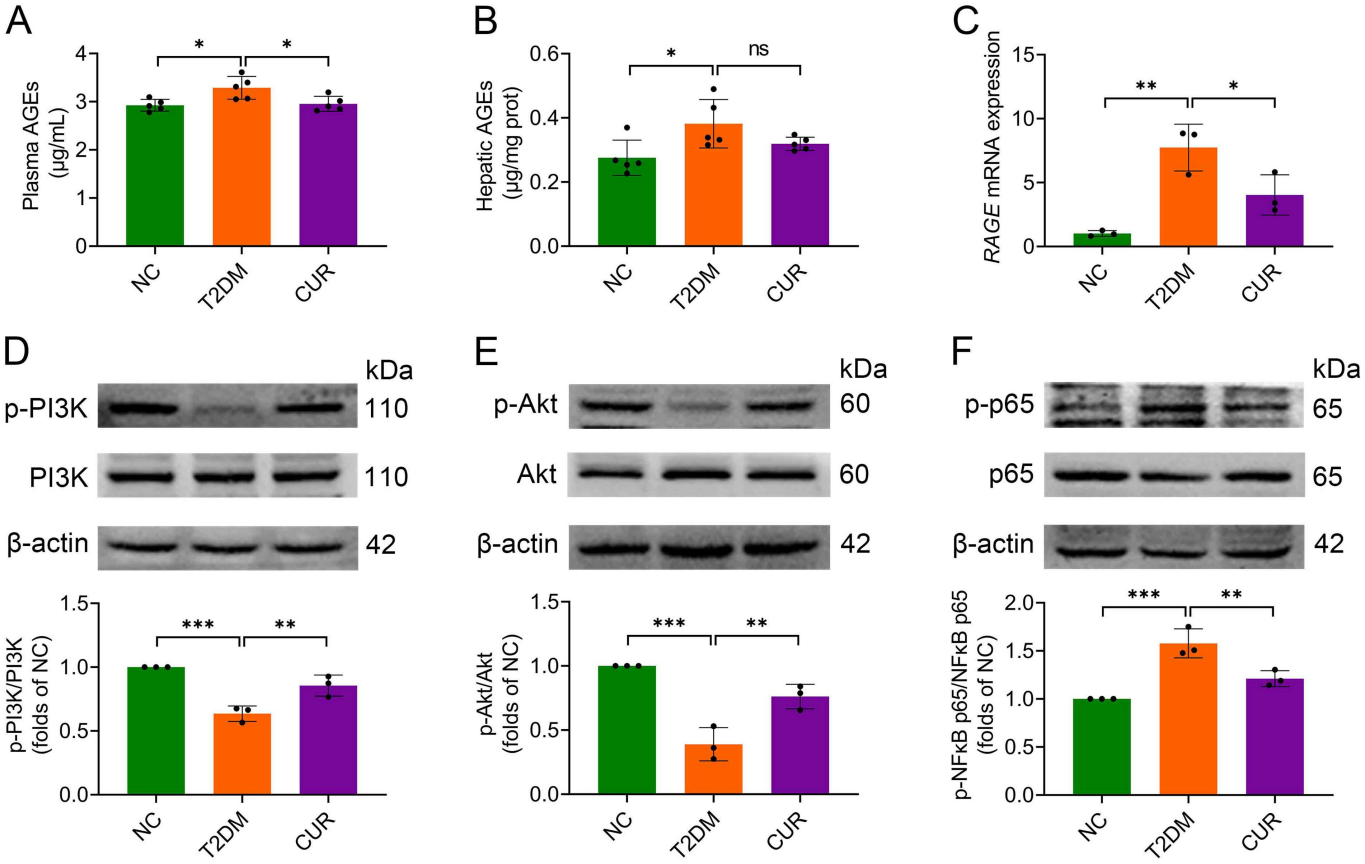
CUR modulates AGE/RAGE signaling and its downstream targets PI3K/Akt and NFκB pathways. **(A,B)** The levels of plasma **(A)** and hepatic AGEs **(B)** were measured by ELISA (*n* = 5). **(C)** The mRNA expression levels of *RAGE* in the liver were analysed (*n* = 3). **(D–F)** Representative bands and quantitative expression levels of p-PI3K, PI3K, p-Akt, Akt, p-NFκB p65, and NFκB p65 proteins in the liver from Western blot were presented. β-actin was served as an internal reference (*n* = 3). For quantitative analysis **(D–F)**, the band intensity of each target protein was normalized to its corresponding internal control. The normalized values were presented relative to the mean of the NC group, which was set to 1. *p < 0.05, **p < 0.01 and ***p < 0.001; ns, not significant difference.

Hyperglycemia-driven AGEs engage hepatic RAGE receptors, promoting oxidative stress and inflammation in hepatocytes (12, 13). Building on the KEGG enrichment results from network pharmacology analysis (Figures 5, 6), we further validated the involvement of this pathway in the effect of CUR on liver injury. As shown in Figures 8A,B, both plasma and hepatic AGE levels were significantly elevated in the T2DM group versus NC controls. CUR supplementation substantially attenuated circulating AGEs, but failed to reduce hepatic AGE accumulation following HFD/STZ administration (Figures 8A,B). Furthermore, hepatic *RAGE* mRNA expression was markedly upregulated in T2DM mice compared to NC group, which was significantly reversed by CUR supplementation (Figure 8C). Given that PI3K/Akt and NFκB signaling act as key downstream effectors of the AGE/RAGE axis (13). We further investigated their involvement in CUR-mediated hepatoprotection. As shown in Figures 8D,E, T2DM mice exhibited significant downregulation of PI3K and Akt phosphorylation in liver tissues relative to controls, which was restored by CUR treatment. Additionally, CUR effectively normalized T2DM-induced phosphorylation of NFκB p65 (Figure 8F). These data collectively demonstrate that CUR supplementation modulates the hepatic AGE–RAGE pathway and its downstream PI3K/Akt and NFκB signaling cascades in T2DM mice.

### 3.7 Untargeted metabolomics analysis

To characterize hepatic metabolic alterations in T2DM, we conducted untargeted metabolomic profiling of liver tissues using UPLC–MS/MS. After filtering out ion features with relative standard deviation (RSD) > 30% in quality control samples, 1,643 and 1,255 secondary metabolites were robustly detected in ESI^+^ and ESI^−^ modes, respectively, across NC, T2DM, and CUR treatment groups. Representative base peak intensity (BPI) chromatograms demonstrating metabolite profiles and analytical reproducibility under both ionization modes are provided in Supplementary Figure S2.

**Figure 9:**
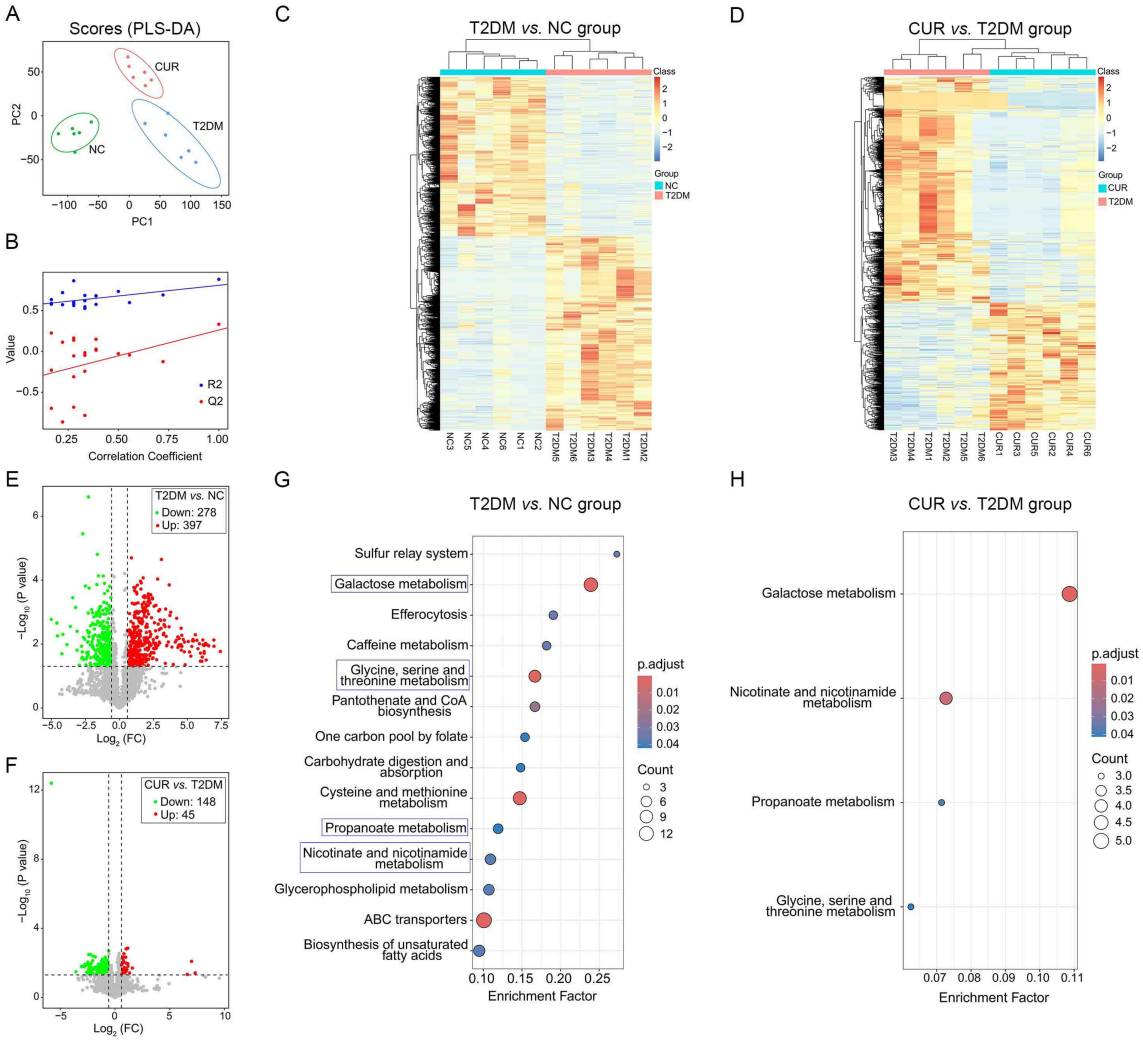
Untargeted metabolomic profiling of liver tissues using UPLC–MS/MS. **(A)** PLS-DA score plot visualizing metabolic distinctions among the three groups. **(B)** *R*^2^*Y* and *Q*^2^*Y* values of the PLS-DA model. **(C,D)** Hierarchical clustering heatmaps of significantly altered metabolites indicating distinct metabolic profiles between comparative groups. **(E,F)** Volcano plots for NC *vs.* T2DM group **(E)**, and T2DM *vs.* CUR group **(F)**. **(G,H)** KEGG enrichment analysis based on differential metabolites for T2DM *vs.* NC group **(G)**, and CUR *vs.* T2DM group **(H)**. Significantly enriched pathways identified via *p*-values and Rich Factor analysis.

To identify differential metabolites, partial least squares-discriminant analysis (PLS-DA) was employed to visualize metabolic distinctions among the three groups. The scores plot revealed distinct clustering patterns; liver samples from NC, T2DM, and CUR groups exhibited clear separation, while intra-group biological replicates demonstrated tight clustering (Figure 9A), indicating robust biological consistency within each cohort. Permutation testing confirmed model validity without overfitting (*R*^2^*Y* = 0.882, *Q*^2^ = 0.333; permutation-derived intercepts: *R*^2^ = 0.549, *Q*^2^ = −0.37) (Figure 9B), demonstrating the model’s explanatory power for metabolic variance. Hierarchically clustered heatmaps of significantly altered metabolites further illustrated distinct metabolic profiles between comparative groups (Figures 9C,D).

To identify significant metabolic alterations, differential metabolites were selected using orthogonal partial least squares-discriminant analysis (OPLS-DA) with variable importance in projection (VIP) > 1.0 and false discovery rate (FDR)-corrected *p* < 0.05. Hierarchical clustering of 720 annotated secondary metabolites (KEGG/HMDB) revealed distinct metabolic profiles across NC, T2DM, and CUR groups. Volcano plots demonstrated that compared to NC controls, T2DM mice exhibited 278 down-regulated and 397 up-regulated hepatic metabolites (Figure 9E). CUR intervention significantly modulated 193 metabolites (148 down-regulated, 45 up-regulated) versus T2DM group (Figure 9F). KEGG pathway analysis of differential metabolites identified significant enrichment in galactose metabolism, glycine/serine/threonine metabolism, propanoate metabolism, and nicotinate/nicotinamide metabolism in T2DM versus NC group (Figure 9G). Notably, CUR supplementation also modulated these metabolic pathways (Figure 9H).

**Figure 10:**
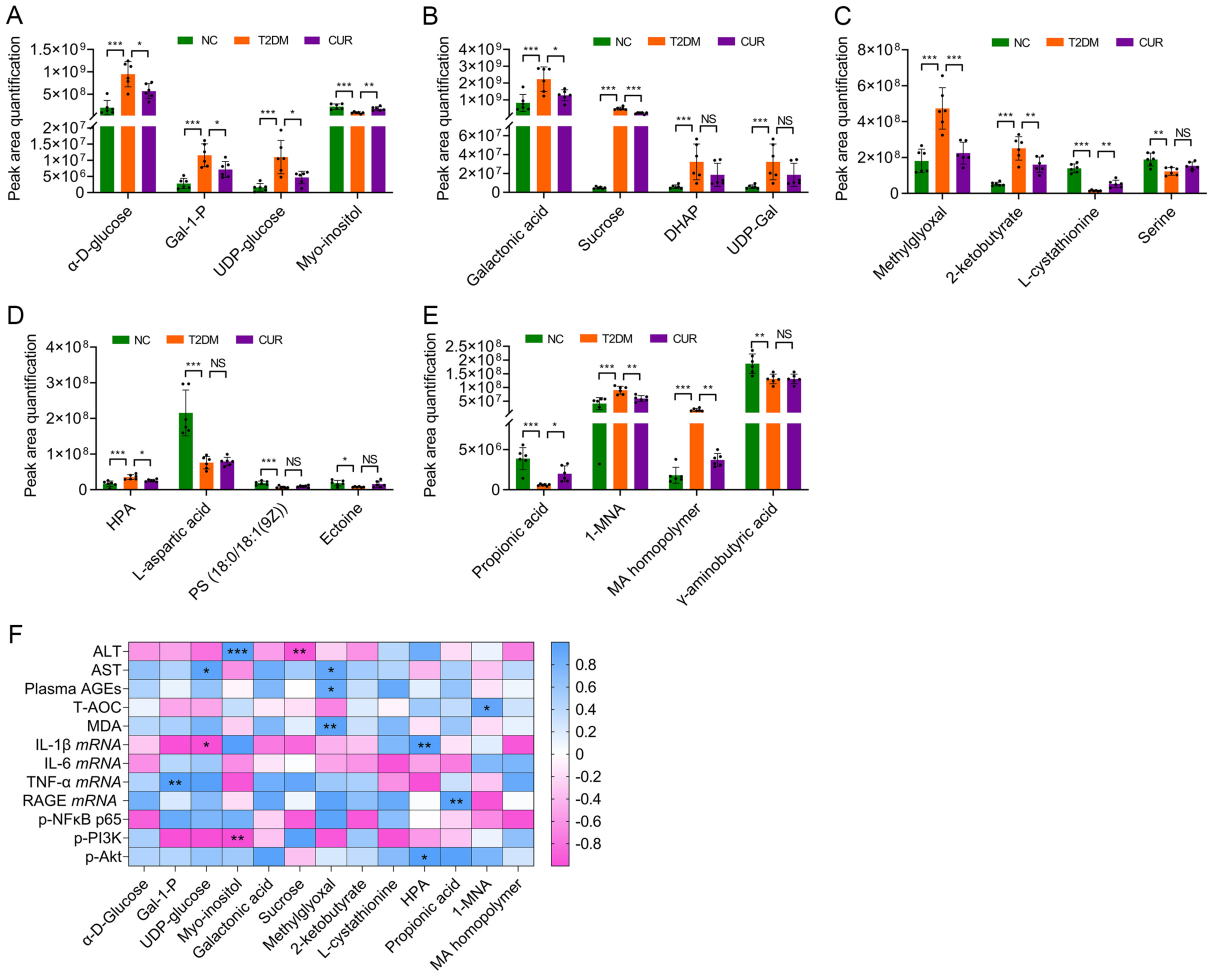
Differential metabolites among the NC, T2DM and CUR groups according to UPLC–MS/MS analysis. **(A–E)** Comparations of hepatic metabolite contents according to their peak area quantification were shown. *p < 0.05, **p < 0.01 and ***p < 0.001; ns, not significant difference (*n* = 6). **(F)** Association between differential metabolites and biological parameters was performed using Pearson’s correlation analysis. *p < 0.05 and **p < 0.01.

Further metabolomic analysis revealed significant perturbations in hepatic galactose metabolism in T2DM mice, characterized by elevated *α*-D-glucose, galactose-1-phosphate (Gal-1-P), uridine diphosphate (UDP)-glucose, galactonic acid, sucrose, dihydroxyacetone phosphate (DHAP), and UDP-galactose (UDP-Gal), alongside reduced myo-inositol levels. CUR supplementation effectively normalized these metabolic alterations (Figures 10A,B). Moreover, disruptions in glycine/serine/threonine metabolism manifested as increased methylglyoxal, 2-ketobutyrate, and hydroxypyruvic acid (HPA), with concomitant decreases in L-cystathionine, serine, L-aspartic acid, phosphatidylserine (18:0/18:1(9Z)), and ectoine (Figures 10C,D). CUR intervention significantly restored methylglyoxal, 2-ketobutyrate, L-cystathionine, and HPA levels (Figures 10C,D). Additionally, propanoate metabolism abnormalities featured reduced propionic acid, which was partially reversed by CUR treatment (Figure 10E). Similarly, nicotinate/nicotinamide metabolism dysregulation showed increased 1-methylnicotinamide (1-MNA) and maleic acid (MA) homopolymer, but decreased *γ*-aminobutyric acid. CUR supplementation significantly modulated 1-MNA and MA homopolymer levels (Figure 10E).

Finally, Spearman correlation analysis was performed to assess the relationships between significantly altered hepatic metabolites and key biological parameters in CUR-treated mice. As shown in Figure 10F, plasma ALT levels exhibited a significant positive correlation with hepatic myo-inositol (*p* < 0.001) and a negative correlation with sucrose (*p* < 0.01). Similarly, plasma AST levels were positively correlated with UDP-glucose and methylglyoxal (both *p* < 0.05). In addition, circulating AGEs (*p* < 0.05) and hepatic MDA (*p* < 0.01) levels both correlated positively with methylglyoxal. Hepatic *IL-1β* mRNA expression was significantly associated with UDP-glucose (*p* < 0.05) and HPA (*p* < 0.01). *TNF-α* mRNA levels exhibited a positive correlation with Gal-1-P concentrations (*p* < 0.01). Moreover, hepatic *RAGE* mRNA expression showed a significant positive correlation with propionic acid (*p* < 0.01). With respect to signaling molecules, PI3K phosphorylation was negatively associated with myo-inositol (*p* < 0.01), whereas Akt phosphorylation was positively correlated with HPA (*p* < 0.05) (Figure 10F).

## 4 Discussion

T2DM is a significant risk factor for liver injury, primarily through the progression of NAFLD and its inflammatory subtype, non-alcoholic steatohepatitis (NASH) (6). Dietary intervention represents a key strategy for preventing and mitigating T2DM progression and associated hepatic complications (19, 33). CUR, a bioactive polyphenol, demonstrates documented hepatoprotective effects in NAFLD (25, 26). However, its mechanisms against diabetic liver injury remain incompletely characterized. Integrating network pharmacology, molecular docking, experimental validation, and untargeted metabolomics, this study elucidated the mechanistic basis of CUR’s hepatoprotection in T2DM mice. We demonstrated that CUR supplementation ameliorated liver injury by attenuating oxidative stress and inflammation, likely through down-regulation of the AGE–RAGE axis and its downstream effectors (PI3K/Akt and NFκB signaling), coupled with modulation of several metabolism pathways. These findings suggest a multi-targeted mechanism underlying CUR’s protection against hyperglycemia-induced hepatic damage.

The progression from T2DM to NAFLD is driven by synergistic perturbations in metabolic signaling (7). Chronic hyperglycemia exacerbates oxidative stress and activates pro-inflammatory cascades, promoting hepatocyte apoptosis and stellate cell activation (34). Furthermore, it is increasingly recognized that such metabolic and inflammatory dysregulation represents a core mechanism of liver injury, applicable not only to endogenous metabolic disorders like diabetes but also to liver damage induced by various exogenous exposures (35). Our current results demonstrated that CUR supplementation attenuated FBG in T2DM mice, consistent with previous reports (36, 37). This glycemic improvement likely contributes to CUR’s attenuation of hepatic oxidative stress and inflammatory injury. Furthermore, impaired hepatic insulin signaling disrupts gluconeogenesis suppression while concurrently activating adipose triglyceride lipase, releasing pathogenic FFAs into systemic circulation (38). Notably, however, 4-week CUR supplementation did not significantly improve glucose intolerance or insulin resistance, aligning with prior studies (39, 40). This indicates that the observed hepatoprotection was likely mediated through more direct mechanisms in the liver. The efficacy of CUR supplementation on glucose intolerance and insulin resistance depends on dosage and duration of intervention.

To elucidate mechanisms underlying CUR’s mitigation of diabetic liver injury, network pharmacology analysis identified AKT1, TNF, TP53, IL-6, and EGFR as top-ranked hub targets against T2DM. Insulin normally regulates metabolism through PI3K/Akt signaling (41). Critically, the impairment of PI3K/Akt signaling disrupts the activation of nuclear factor erythroid 2-related factor 2 (Nrf2), a master transcription factor that orchestrates the expression of a wide array of cytoprotective and antioxidant genes and plays a pivotal role in cellular defense systems (42, 43). Therefore, the subsequent PI3K/Akt impairment suppresses this vital Nrf2-mediated antioxidant defenses while concurrently activating NFκB-driven inflammation, thereby exacerbating mitochondrial ROS production and cytokine release (e.g., IL-6, TNF-*α*, IL-1β) in diabetic complications (41, 44). Crucially, our data demonstrate that CUR supplementation significantly activated PI3K/Akt signaling while suppressing NFκB during HFD/STZ treatment. Moreover, as elevated hepatic p53 correlates with hyperglycemia and HOMA-IR in T2DM patients and impairs insulin sensitivity (45), we observed that CUR treatment markedly reduced T2DM-induced p53 phosphorylation. Molecular docking confirmed favorable binding affinities between CUR and AKT1/TP53. Collectively, CUR’s modulation of PI3K/Akt, NFκB, and p53 pathways likely contributes to attenuated diabetic liver injury, though crosstalk mechanisms warrant further investigation.

AGEs represent key mediators in diabetes progression and its complications. Under normoglycemic conditions, AGEs form at moderate rates, but persistent hyperglycemia markedly accelerates their accumulation due to increased glucose availability (15). Engagement of the RAGE sustains cellular dysfunction and critically contributes to diabetes pathogenesis during chronic hyperglycemia (13). The AGE–RAGE axis exacerbates NAFLD by orchestrating oxidative stress, inflammation, and fibrogenesis (46). In insulin-resistant states, PI3K/Akt and NFκB pathways serve as major downstream effectors of AGE–RAGE signaling. RAGE ligation inactivates PI3K/Akt signaling, paradoxically impairing insulin transduction through IRS-1 serine phosphorylation, thereby exacerbating insulin resistance and *de novo* lipogenesis (47). Concurrently, RAGE activates NFκB via IκB*α* degradation, driving pro-inflammatory cytokine transcription and oxidative stress that perpetuate hepatocyte injury (48). NFκB activation further upregulates RAGE expression, amplifying AGE-induced damage in a feedforward loop (49). This cascade suppresses Nrf2-mediated antioxidant defenses (via impaired Akt-dependent Nrf2 nuclear translocation) and activates hepatic stellate cells (HSCs), promoting collagen deposition (50). Thus, pharmacological interventions targeting the AGE–RAGE axis can restore PI3K/Akt homeostasis while suppressing NFκB, offering dual therapeutic potential against diabetic hepatopathy (51).

Our current study demonstrated that CUR supplementation greatly downregulated AGE–RAGE axis in T2DM mice by reducing plasma and hepatic AGE accumulation and attenuating hepatic *RAGE* mRNA expression. Molecular docking confirmed favorable binding affinity between CUR and RAGE. CUR-mediated disruption of AGE–RAGE signaling likely contributes to PI3K/Akt pathway activation and NFκB suppression, thereby alleviating hepatic oxidative stress and inflammation in T2DM. Given the multifactorial nature of diabetic liver injury, further elucidation of CUR’s multi-target networks under hyperglycemic conditions is warranted to fully establish its preventive potential. Furthermore, to directly validate the specific involvement of the AGE/RAGE axis proposed in this study, future research employing genetic approaches, such as the use of RAGE knockout animal models or siRNA-mediated gene silencing in human hepatocyte lines (e.g., HepG2), will be essential to conclusively establish the causal role of this pathway in CUR-mediated protection against diabetic liver injury.

The core pathophysiological features of T2DM include chronic hyperglycemia and dysregulated lipid/protein metabolism. While prior studies established curcumin’s (CUR) capacity to ameliorate diabetic glycolipid disorders (36, 37), the underlying mechanisms remained incompletely characterized. Metabolic dysregulation constitutes a primary driver of T2DM-induced hepatic impairment (6). Our metabolomic analysis revealed aberrant galactose metabolism in T2DM liver tissue, characterized by elevated levels of α-D-glucose, galactonic acid, Gal-1-P, sucrose, and related intermediates. This aligns with reports implicating specific galactose pathway metabolites in diabetic hepatopathy. α-D-glucose directly contributes to hyperglycemia-induced liver injury (52). Gal-1-P accumulation (due to Gal-1-P uridylyltransferase (GALT) deficiency) exacerbates insulin resistance and hepatotoxicity via disrupted Leloir pathway flux (53). UDP-glucose accumulaion induced by inhibited activation of UDP-glucose 6-dehydrogenase (UGDH) hastens the development of NASH-associated liver damage and fibrosis (54). Exogenous galactose sources (e.g., sucrose) increase the ability of the liver to produce lipids (55). CUR supplementation significantly reduced these pathogenic metabolites, suggesting its modulation of galactose metabolism may disrupt oxidative-inflammatory crosstalk in diabetic hepatopathy. Notably, CUR also reversed T2DM-induced depletion of hepatic myo-inositol, a metabolite with established protective roles in diabetes (56), further supporting its hepatoprotective potential.

Furthermore, hepatic amino acid metabolism dysregulation has been documented in diabetic db/db mice (57). Our study corroborates this observation, demonstrating significant disruption of glycine/serine/threonine metabolism in T2DM liver tissue. This metabolic perturbation manifested as elevated methylglyoxal and 2-ketobutyrate alongside reduced L-cystathionine levels. Such dysregulation exacerbates diabetic hepatopathy through redox imbalance and inflammatory signaling (58). Mechanistically, methylglyoxal, as a dicarbonyl metabolite of glucose, is increased in hyperglycemic conditions and can modify proteins with arginine, cysteine, and lysine residues to produce AGEs (59). Its accumulation induces hepatotoxicity via ROS-mediated mitochondrial dysfunction and enhanced inflammation (60). 2-ketobutyrate derived from threonine catabolism inhibits mitochondrial pyruvate dehydrogenase, impairing acetyl-CoA generation and promoting lipid peroxidation via ROS overproduction (61). Conversely, L-cystathionine demonstrates antioxidant properties and protects against DNA damage (62). CUR supplementation significantly normalized these metabolic alterations in the liver of T2DM mice by decreasing methylglyoxal and 2-ketobutyrate, and increasing propionic acid and L-cystathionine. This coordinated metabolic reprogramming likely attenuates oxidative stress and inflammatory injury in diabetic hepatopathy.

Methylglyoxal, 2-ketobutyrate, and HPA share the common intermediates within propanoate and amino acid metabolic pathways. Elevated levels of methylglyoxal, 2-ketobutyrate, and HPA, coupled with reduced propionic acid, indicate dysregulation of propanoate metabolism—a perturbation implicated in diabetic hepatopathy through mitochondrial dysfunction and inflammatory signaling (63, 64). Furthermore, 1-MNA is a methylated derivative of nicotinamide and serves as a key intermediate in the nicotinate/nicotinamide metabolism. While increased 1-MNA enhances NAD^+^ salvage pathways to mitigate mitochondrial oxidative stress, it may paradoxically compromise sirtuin-1 activity, exacerbating insulin resistance and *de novo* lipogenesis (65). CUR supplementation significantly attenuated T2DM-induced accumulation of methylglyoxal, 2-ketobutyrate, HPA, and 1-MNA, indicating its regulatory effects on both propanoate metabolism and nicotinate/nicotinamide metabolism. This metabolic normalization likely contributes to CUR’s mitigation of diabetic hepatopathy. Finally, observed correlations between differential metabolites and biological parameters in CUR-treated mice suggest metabolic pathway modulation as a key mechanism underlying CUR’s efficacy. Future studies should elucidate the direct relationship between AGE–RAGE signaling and T2DM metabolic dysregulation under CUR intervention.

While our study demonstrated the promising efficacy of CUR (800 mg/kg diet) in alleviating diabetic liver injury in mice, several aspects warrant careful consideration to contextualize these findings and guide future research. A well-recognized paradox in CUR pharmacology is its considerable bioactivity despite low systemic bioavailability, a phenomenon increasingly attributed to the actions of its bioactive metabolites, particularly tetrahydrocurcumin (THC) (66). Growing evidence indicates that THC may possess superior antioxidant and anti-inflammatory potency compared to the parent compound (67), suggesting that the hepatoprotective effects observed here may be mediated at least partially via this metabolite. When translating these results to a clinical context, it is important to note that the murine dose used in this study (equivalent to ~100 to 150 mg/kg/day) substantially exceeds typical human supplemental doses, which generally range from 0.25 to 1.0 g/day (~4.2 to 16.7 mg/kg for a 60 kg adult) and have nonetheless shown beneficial effects on liver health markers in clinical settings (68, 69). Another limitation of the present work is the use of a single dose, which prevents evaluation of potential dose–response relationships. To systematically bridge these preclinical results to clinical applications, future studies should prioritize: (1) direct comparative analyses of CUR and THC to identify the more potent therapeutic entity; (2) dose-ranging experiments to establish an optimal dosing regimen; and (3) well-designed clinical trials involving diabetic patients with confirmed liver injury, to evaluate the therapeutic potential of CUR or its optimized formulations.

## 5 Conclusion

Our findings elucidate a novel mechanism by which CUR supplementation ameliorates diabetic liver injury in T2DM. CUR effectively attenuated T2DM-induced hepatopathy by mitigating oxidative stress and inflammation, primarily through inhibition of the AGE–RAGE signaling pathway and restoration of metabolic homeostasis. These results highlight CUR’s potential for preventing diabetic liver injury and provide new insights for clinical translation.

## Data Availability

The datasets presented in this study can be found in online repositories. The names of the repository/repositories and accession number(s) can be found in the article/Supplementary material.

## Footnotes

^1^https://pubchem.ncbi.nlm.nih.gov/, updated 2024.

^2^http://www.swisstargetprediction.ch, updated 2024.

^3^http://targetnet.scbdd.com/, updated November 26, 2024.

^4^https://prediction.charite.de/, updated March 2022.

^6^https://omim.org/, updated November 2024.

^7^http://db.idrblab.net/ttd/, updated January 10, 2024.

^8^https://www.genecards.org/, Version 5.22, updated October 9, 2024.

^9^https://www.pharmgkb.org/, accessed November 27, 2024.

^10^http://www.malacards.org/, Version 5.22.

^11^http://www.ehbio.com/test/venn/, updated November 30, 2024.

^12^http://www.cytoscape.org/, version 3.9.1.

^13^https://www.rcsb.org/, accessed on February 20, 2025.

^14^Version 3.1.0; https://openbabel.org/.

## Glossary

AGE: advanced glycation end product
ALT: alanine aminotransferase
ANOVA: analysis of variance
AST: aspartate aminotransferase
AUC: area under the curve
CUR: curcumin
DM: diabetes mellitus
FBG: fasting blood glucose
FFAs: free fatty acids
GSH: glutathione
IL-1β: interleukin-1β
IR: insulin resistance
ITT: insulin tolerance test
MDA: malondialdehyde
NAFLD: nonalcoholic fatty liver disease
NASH: nonalcoholic steatohepatitis
OGTT: oral glucose tolerance test
RAGE: receptor for advanced glycation end product
ROS: reactive oxygen species
SOD: superoxide dismutase
STZ: streptozotocin
T-AOC: total antioxidant capacity
TNF-*α*: tumor necrosis factor-α
TP53: tumor protein p53
T2DM: type 2 diabetes mellitus
UPLC–MS/MS: ultra-performance liquid chromatography–tandem mass spectrometry
