# Supplementary material for: Curcumin attenuates liver injury by modulating the AGE–RAGE axis and metabolic homeostasis in high-fat diet/streptozotocin-induced type 2 diabetic mice
Source: Front Nutr. 2025 Nov 7;12:1710380. doi: 10.3389/fnut.2025.1710380 (PMC12635622; doi:10.3389/fnut.2025.1710380)
Supplement: Supplementary file 1 [file Data_Sheet_1.PDF]

Supplementary Material

Curcumin Attenuates Liver Injury by Modulating the AGE–RAGE Axis and Metabolic Homeostasis in High-Fat Diet/Streptozotocin-Induced Type 2 Diabetic Mice

Mengyao Li[1#], Chunmei Zhang[2#], Junyu Ma[1], Bangzhao Zeng[1], Xuexun Li[1], Xin Zhao[1], Xiaoyan Bi[1], Rong Li[1], Qin Gao[3], Yang Jiang[1*], Fuli Ya[1*]

[1]*Department of Nutrition, School of Public Health, Dali University, Dali, Yunnan Province 671000, PR China;*

[2]*Department of Laboratory Teaching Center, School of Public Health, Dali University, Dali, Yunnan Province 671000, PR China;*

[3]*School of Public Health, Jining Medical University, Jining, Shandong Province 272000, PR China*

[#]Mengyao Li and Chunmei Zhang contributed equally to this work.

*Corresponding authors:
Fuli Ya, MD, Ph.D
Department of Nutrition, School of Public Health, Dali University,
No. 22, Wanhua Road, Dali, Yunnan Province, PR China 671000;
E-mail: yafuli@yeah.net; Tel/Fax: 86-872-2257382

Yang Jiang, MD, Ph.D
Department of Nutrition, School of Public Health, Dali University,
No. 22, Wanhua Road, Dali, Yunnan Province, PR China 671000;
E-mail: yangjiang@dali.edu.cn

Supplementary Table S1. The feed composition for animal diets.

Ingredient	Low-fat diet: g (kcal)	High-fat diet: g (kcal)
Casein	200 (800)	200 (800)
Corn starch	452.2 (1808.8)	72.8 (291)
Maltodextrin	75 (300)	100 (400)
Sucrose	172.8 (691.2)	172.8 (691)
Soybean oil	25 (225)	25 (225)
Cellulose	50 (0)	50 (0)
Mineral mix	45 (0)	45 (0)
Vitamin mix	10 (40)	10 (40)
L-cystine	3 (12)	3 (12)
Choline bitartrate	2 (0)	2 (0)
Lard	20 (180)	177.5 (1598)
Total	1050 (4057)	858.1 (4057)

Supplementary Table S2. Primer sequences used for qRT-PCR.

Genes	Primer	Sequence
IL-1β	Forward	5'-TGGACCTTCCAGGATGAGGACA-3'
	Reverse	5'-GTTCATCTCGGAGCCTGTAGTG-3'
IL-6	Forward	5'-TACCACTTCACAAGTCGGAGGC-3'
	Reverse	5'-CTGCAAGTGCATCATCGTTGTTC-3'
TNFα	Forward	5'-GGTGCCTATGTCTCAGCCTCTT-3'
	Reverse	5'-GCCATAGAACTGATGAGAGGGAG-3'
RAGE	Forward	5'-GCCACTGGAATTGTCGATGAGG-3'
	Reverse	5'-GCTGTGAGTTCAGAGGCAGGAT-3'
β-actin	Forward	5'-CATTGCTGACAGGATGCAGAAGG-3'
	Reverse	5'-TGCTGGAAGGTGGACAGTGAGG-3'

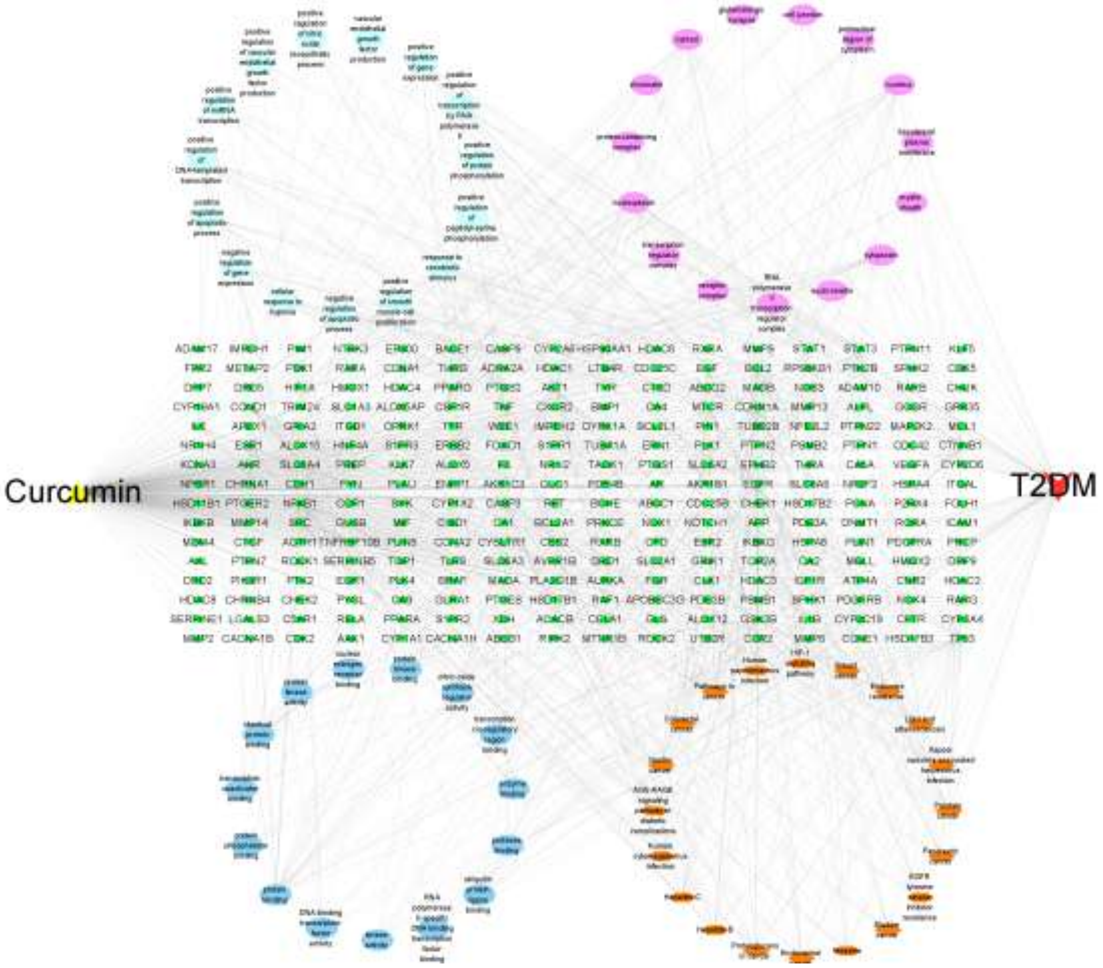

Supplementary Fig. S1. A tripartite network integrating Curcumin (CUR), predicted targets, and enriched pathways was constructed. Nodes represent molecular entities while edges denote biological associations, enabling systematic prediction of CUR's therapeutic mechanisms in T2DM.

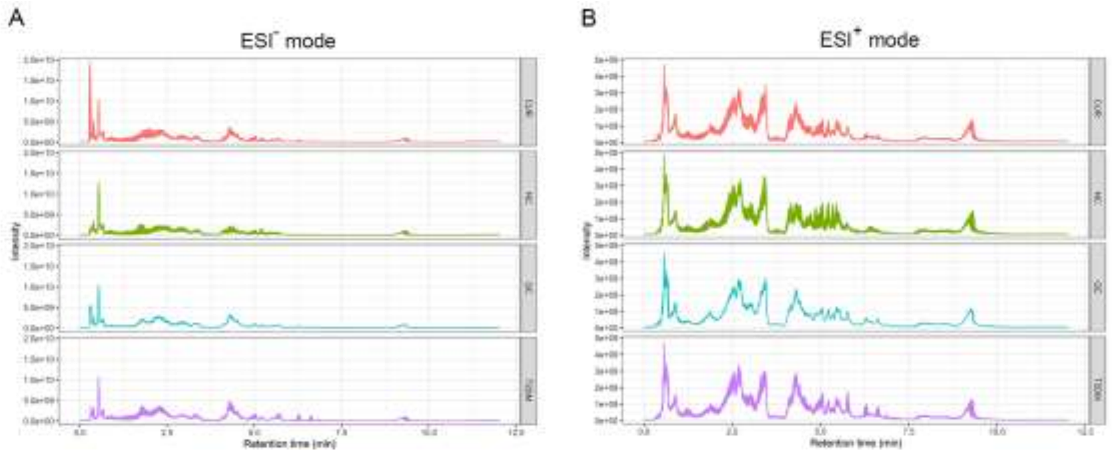

Supplementary Figure S2. The basic peak intensity chromatogram of lipids among NC, T2DM and CUR groups as well as the quality control (QC) in ESI^- and ESI^+ modes were shown.
